# Australian primary care policy in 2004: two tiers or one for Medicare?

**DOI:** 10.1186/1743-8462-1-2

**Published:** 2004-11-17

**Authors:** Hal Swerissen

**Affiliations:** 1 Director, Australian Institute for Primary Care La Trobe University Australia

## Abstract

The recent primary care policy debate in Australia has centred on access to primary medical (general practice) services. In Australia, access is heavily influenced by Commonwealth Government patient rebates that provide incentives for general practitioners not to charge copayments to patients (bulk billing). A steady decline in key access indicators (bulk billing) has led the Howard Government to introduce a set of changes that move Medicare from a universal scheme, to one increasingly targeted at providing services to more disadvantaged Australians. In doing so, another scene in the story of the contest between universal health care and selective provision in Australia has been written. This paper explores the immediate antecedents and consequences of the changes and sets them in the broader context of policy development for primary care in Australia.

## Introduction

Primary health care and community care can be thought of as a set of health programs and services. Most discussions of the primary health and community care services sector suggest that it has the following characteristics: (1) It is the first point of contact with the health system. This may occur through general practice, community health services, and pharmacies. There is also some overlap between primary care and hospital emergency departments, particularly for less complex and intensive presentations. (2) Services are provided in community and ambulatory settings and at home. (3) There is an emphasis on continuing relationships between service providers and consumers over extended periods of time. (4) Services have a more comprehensive and holistic approach. (5) There is an emphasis on early detection and illness prevention services such as maternal and child health programs and population health programs including health promotion.

Primary health and community care is the most visible and commonly used part of the health system. In 1999–00 the Commonwealth provided approximately $6 billion through the Commonwealth Medical Benefits and Pharmaceutical Benefits Schemes. States and Local Government provided approximately $1.8 billion for 'community and public health' which includes allied health, counselling, nursing and a range of primary and secondary prevention and health promotion programs. The Commonwealth, through direct outlays ($6 million) and private health insurance premium rebates ($97 million), also provided $103 million for dental services, with the States and Territories contributing $305 million. This does not include the substantial funds committed to the various forms of community support for people with disabilities, chronic illness and mental illness [1].

Primary health and community care services face unique challenges. Over the past three decades primary health services have come under significant pressure to address a more complex and diverse range of community needs.

Deinstitutionalisation, the introduction of new health and information technologies, the increasing prevalence of chronic disease and more general social and economic trends have had a significant impact on primary health and community support services. This has resulted in concerns about the equity, quality and efficiency of services and programs. Arguably, there is a need for a national primary health care policy in Australia. One that would address system integration, care pathways and team practice, work force development, payment arrangements, governance, performance management and accountability. However, current Commonwealth reforms are focused on important, but relatively, narrowly focused solutions to the decreasing affordability and access for general medical services. This article focuses primarily on the recent debate that surrounds this issue.

### Recent Policy

Medicare is a Commonwealth Government, tax funded, social insurance scheme that provides rebates for general (primary) and specialist medical services and optometry. In Australia, it is the principal national program for ensuring equitable access to primary medical services. Over the last two years there has been a fiercely contested debate about the future of Medicare.

Medicare was introduced to provide universal access to affordable medical care. Up until recently Medicare simply provided a rebate of 85% of the Commonwealth Government determined schedule fee for medical and diagnostic services. Practitioners were free to charge patients a copayment as well. Where they did not apply a copayment, they could bill the Commonwealth for the rebate and receive bulk payments direct from the Commonwealth for these services, thereby avoiding administrative costs and delay. This payment method, which became known as bulk billing, ensured that services were effectively free to the patient at the point of service.

Medicare has been very successful, particularly for general practice services. It is strongly supported by the Australia community because it provides affordable access to medical services. Despite historical resistance by the Australian Medical Association, it has been widely supported by practitioners because it provides them with a universal, simple and predictable revenue stream.

Bulk billing increased steadily from the introduction of Medicare in 1984/85 to approximately 70% in the mid-1990s. Bulk-billing rates for GP services have generally been about 10% higher than the overall bulk-billing rates over the last decade, reaching a plateau of about 80% in the mid-1990s. They declined significantly after 2000. Average GP bulk billing fell to 68% by September 2003. GP bulk billing rates are now similar to the overall CMBS bulk billing rates for all services [2].

As bulk billing rates declined, disquiet and concern about access to medical services rose amongst stakeholder interests. More generally, the overall decline in bulk billing came on top of considerable disparity in equity of access between rural and urban settings. Bulk billing rates in inner city areas with high per capita GP ratios were 30% higher than those in rural settings with low per capita ratios. A number of remote rural areas had difficulty attracting any GPs at all.

Analysis of the reasons for the decline in bulk billing and the disparities between rural and metropolitan settings suggest a strong relationship between the supply of GPs and the capacity to charge copayments and the importance of the steady decline in the relative value of Commonwealth rebates for GP services over time.

There is considerable evidence that GPs manage demand for their services to maintain their income [3]. As the number of GPs increased with introduction of Medicare, particularly in inner city areas, per capita utilisation of GP services increased sharply. Average out of pocket costs for patients fell as bulk billing increased. The Commonwealth effectively provided the 'floor price' for services in areas of high supply and high competition leaving patient throughput rates as the primary means for increasing revenue. Urban areas with greater levels of disadvantage had higher bulk billing rates and shorter consultation times. In higher socio-economic status areas, where patients have a greater capacity to pay, there were lower bulk billing rates and longer consultation times.

In the decade to 2003, changes to GP training, migration and demographic ageing lead to a stabilization and decline in the supply of GP services. Over the same period, the relative value of Medicare rebate income for GP services fell by about 10 percent compared with average weekly ordinary time earnings. A decline that was probably even greater when compared to specialist incomes. In response, GPs began to experiment with price increases (co-payments) to improve their relative incomes [4].

Interestingly, bulk billing rates in relatively under supplied rural settings remained relatively stable at about 50% of consultations. This is about the level of consultations one would expect people on low incomes, who are eligible for concessional welfare benefits, to use. A finding that suggests that patient capacity to pay sets a 'floor' bulk billing rate at about this level [4].

It is worth noting that many of these effects were predictable from the reforms to General Practice introduced in the early 1990s. In particular, tight management of GP supply through changes to training programs and restrictions on overseas trained medical practitioners were introduced in order to reduce growth in aggregate Medicare expenditure. However, the reforms recognised that a move away from fee for service payment would also be required in the longer term. To this end a Better Practice Program to pay GPs on a per capita basis was introduced.

Over time, it was intended that a significant proportion of Medicare payments would be made by practice based, per capita payments. Progressively this would have allowed a shift toward more comprehensive, integrated practice and a greater focus on quality and preventive services. However, while the supply of GPs was successfully constrained, per capita payments remained a marginal component of the payment system.

In response to concerns about the fall in the bulk billing rate, the Commonwealth Government proposed a "Fairer Medicare" package in April 2003. The package introduced a participating practice scheme. GP practices that agreed to charge a no gap fee to concessional patients were to be eligible for increased Medicare rebates for these patients. The level of the proposed increase for the rebate was $1 in metropolitan city practices, $2.95 in non-metropolitan city practices, $5.30 in rural centre practices, and $6.30 in outer rural and remote areas.

Participating practices were to continue to have the capacity to determine fees for non-concession cardholders, including the option of bulk billing. However, if they chose not to bulk bill these patients, they were to be able to charge the patient the co-payment and claim the Medicare rebate direct from the Health Insurance Commission through HIC online billing facilities. Effectively, non-concession cardholders were to be charged a gap payment thereby avoiding the transaction costs involved in claiming a rebate through the Medicare scheme themselves.

A new MBS safety net was to be available for those covered by concession cards with out-of-pocket costs greater than $500 in a calendar year. Charges in excess of the scheduled fee were to be included, as were the costs of specialist and diagnostic services. Eighty per cent of out-of-pocket costs above the $500 threshold were to be met through this safety net.

Private health insurers were to be able to offer insurance coverage for the cumulative cost of out-of-hospital medical services over $1,000 for a family in a calendar year. This included costs above the scheduled fee across a range of out-of-hospital services, including GP and specialist consultations and diagnostic tests. The Commonwealth estimated that insurance products for this coverage were likely to cost around $50 per year for families, and the 30% private health insurance rebate was to apply to these products.

The Government's package also included proposals to introduce additional medical school places, additional GP training places, additional nurses and allied health professionals in general practice, and measures for veterans.

The Fairer Medicare package resulted in considerable debate and criticism, much of which was considered by the Senate Select Committee on Medicare [5]. In part the Committee concluded that:

• Equitable access to general practice services regardless of income or geography is fundamental to good health care.

• GP income from bulk billing had not kept pace with increases in average weekly ordinary time earnings and this had contributed to declining bulk billing rates and increased out of pocket charges.

• Shortages in the supply of GPs are emerging as result of compositional changes in the workforce, changes in practice patterns and population ageing.

• The Commonwealth's 'Fairer Medicare' proposals were inconsistent with the principles of Medicare.

• The differential rebate payments for concessional patients were unnecessary because these patients were already largely receiving bulk billed services

• The introduction of the new safety net arrangements creates a two tier system of access to GP services

It became apparent that the Senate would not pass the legislation required to enact the Commonwealth's package. Consequently, the Commonwealth presented its 'MedicarePlus' extensions and revisions to the original proposal in November 2003 [6]. This package was passed by the Australian Senate with the support of four independent Senators.

The MedicarePlus proposals dropped the participating practice scheme. Instead the Commonwealth proposed to increase the rebate for all concessional patients by $5 in metropolitan areas and $7.50 in remote, rural and regional areas (including the State of Tasmania). The increased rebate was also extended to children under 16. The safety net provisions were modified to provide an 80% rebate for out of hospital medical costs for concessional patients and those whose income fell below specified tax thresholds after $300 of out of pocket expenses and after $700 for the remainder of the population.

Other aspects of the original proposal were largely retained and extended. These included training places for GPs, medical graduates and nurses. Additionally, it was proposed to introduce a Medicare Benefits Schedule item for nursing support in general practice and improved internet access and online billing for GPs. MedicarePlus also provides rebates for up to five allied health consultations delivered to patients with a chronic condition or complex care needs, for and on behalf of a GP. Similarly, dental treatment care plans will be funded for these patients where they have significant dental problems that exacerbate their condition. The total estimated cost for MedicarePlus to 2006/07 was estimated at $2.85 billion.

### Policy Analysis

Initial reactions to the Commonwealth's proposals were mixed. A number of patient and provider groups have criticized the new arrangements as undermining the principle of universality that underpins Medicare. Criticisms have also focused on the narrow focus of MedicarePlus on fees for general practitioners.

More specifically, MedicarePlus is likely to have differential effects on affordability and access to GP services in rural and metropolitan settings. In metropolitan settings, the introduction of a $5 differential rebate for bulk billing concessional payments is sufficient to increase net GP incomes to about the AWOTE relativities that applied prior to the decline in bulk billing.

However, with current levels of bulk billing still at over 65% in metropolitan areas, virtually all concessional patients are already bulk billed. The proposal is therefore subject to substantial dead weight loss. No incentives to bulk bill non concessional patients (other than non concessional children aged less than 16) are included.

In metropolitan areas, the gap between the average Medicare rebate and the average patient billed service is around $13 for patients not covered by the differential rebate, compared to $8 for concessional patients and children under 16. Within system constraints, GP incomes are optimized by bulk billing concessional patients and children under 16 and charging copayments for other patients. Doing so also largely addresses patient capacity to pay issues. In the absence of major changes to supply or GP costs, it is therefore likely that bulk billing rates in metropolitan areas will continue to decline over time, until they stabilise at around the level of services for concessional patients plus non concessional children under 16 (around 60% in metropolitan areas, assuming children under 16 have average population consultation rates).

The proportion of children is highest in outer metropolitan regions and lowest in the inner city. Bulk billing rates have declined most in outer metropolitan areas. It is therefore plausible that this measure will have the greatest differential impact in outer metropolitan regions.

In rural settings, where there are GP shortages, the differential rebate (which is higher than in metropolitan settings) could substantially increase GP incomes. However, GP supply factors ensure GPs have considerable capacity to increase copayments within the limits of patient capacity to pay. In general, bulk billing rates in rural settings are now at or below the consultation rate for concessional patients. The new arrangements are therefore likely to protect bulk billing rate for concessional patients and are likely to see the rate increase to the level of concessional consultations (around 55 – 60%).

The effect of differential rebates for non concessional children under 16 on overall bulk billing rates is less clear in rural settings. With the increased rebate, there remains a gap of approximately $5.50 between the new rebate and the average patient billed service. As for metropolitan settings, there are no additional incentives to bulk bill other non concessional patients. Given the greater capacity to charge copayments, this measure may be less successful in encouraging bulk billing than in metropolitan areas.

The safety net provisions in MedicarePlus have significant inflationary potential for out of hospital medical service fees. Concessional patients and those who qualify for Family Tax Benefit A are eligible for an 80% rebate on out of hospital costs once they incur $300 of out of pocket costs. There is no cap on the rebate under the safety net. Average out of pocket costs for patient billed GP services are currently about $13. The safety net is therefore reached in 20–25 consultations. The safety net provisions will be invoked more quickly when specialist medical practitioners, diagnostic imaging services and pathology are required. Average copayments are two or three times higher for specialist medical practitioners than for GPs.

Effectively, the introduction of the safety net removes constraints on medical practitioners associated with concerns about patient capacity to pay. This introduces moral hazard for practitioners and consumers. Practitioners have incentives to increase their fees and provide more services than necessary knowing the safety net will protect patients. Patients have incentives to consume more services than are necessary because they are effectively insured by the safety net.

However, the initial threshold and value of copayments act as balancing disincentives for utilization. Clearly if the initial threshold and the copayments were less, the hazard would be greater and vice versa. This trade off is likely to impact differently depending on need, capacity to pay and supply factors.

For example, there may be paradoxical adverse effects for patients with significant ongoing health costs who are currently bulk billed because GPs and specialists have concerns about their capacity to meet aggregate out of pocket costs over time. This is particularly true for aged pension recipients with chronic illness. With the introduction of the safety net, the potential for incurring unmanageable costs is significantly reduced and therefore bulk billing rates for this group may decline. Whether effects like these are experienced in practice will depend on factors such as the real value of GP rebates, patient need, capacity to pay, GP supply and regulatory constraints.

Overall, the design features of the Howard Government recent changes to Medicare are intended to, and will produce a two tier system. Access to primary medical services for people on low incomes will be relatively well protected, but those above the income threshold will see a steady decline in bulk billing and an increase in out of pocket costs for these services. Additionally, the poorly designed safety net will have inflationary consequences.

### Future Directions

The policy and political contest around Medicare has an extended pedigree. The conservative Liberal/National Party Coalition has long held the position that government should primarily provide health services for those who are unable to provide for themselves and that those who are able to make their own way should do so, particularly by taking out private health insurance. From this perspective the role of government is to provide an appropriate regulatory environment, incentives and sanctions to take up private insurance and a targeted safety net for the disadvantaged. Their preferred model was developed and refined in the 1950s and 1960s during the period of the Menzies Government and reintroduced in stages during the late 1970s and early 1980s by the Fraser Government [7].

On the other hand, the Australian Labor Party has advocated tax funded, universal access to publicly funded health care provided on the basis of need, rather than capacity to pay. The Whitlam Government settled the basic architecture of Labor's approach in the early 1970s. Setting aside the brief flirtation with a national community health program for primary care, it established a universal system of public hospital access through the States and a tax funded, social insurance scheme to underwrite equitable access to medical and related services.

Notwithstanding Howard Government claims of strong support for Medicare, the pendulum has now swung a considerable distance back toward the traditional Liberal/National Party preferred model. If history is a guide, now that the incentives to take out private health insurance and the safety net is in place, the next steps are regulatory mechanisms to exclude higher income earners from accessing publicly funded health services.

While debates about access and equity are critical, they are only part of the overall picture. Recontesting the basic access and equity principles of the health system every decade or so misses a number of important emerging issues.

There is now emerging evidence that closer integration of clinical decision-making and purchasing for enrolled populations in primary care settings through funds pooling and local agreements and contracts has the potential to increase innovation, reduce costs and improve outcomes. These principles are being explored or actively implemented in a number of countries comparable to Australia, including the United Kingdom and New Zealand [8].

There is clearly a need to reconsider the development of a national policy for primary health and community support services. Such a policy might include the following elements to address the issues which have been discussed above:

• National primary health and community care goals and objectives. For example, these might broadly set out equity, efficiency and quality criteria for the Australian primary health and community support system.

• National performance indicators. For example, these indicators could be used to report on and benchmark the quality, access, efficiency and utilisation of the primary health and community support system and its impact on acute, sub acute and residential care.

• Population based planning, allocation and monitoring. For example, funding allocation models and system governance arrangements based on the health care needs of geographically defined residential populations (e.g. Divisions of General Practice, Area Health Authorities, Districts, Primary Care Partnerships) that promote continuity of care and service integration could be considered.

• Coordinated service pathways for health issues and conditions. For example, consistent best practice models linking prevention, early intervention, primary care, acute care, rehabilitation and community support should be developed for all major chronic diseases, mental illness and alcohol and drug problems.

• Payment systems. For example a program to develop integrated payment models and systems for primary and community support services could be established and linked to Commonwealth/State agreements (e.g. AHCAS, HACC) and own purpose funding streams. This might include consideration of capitated, case based, and contract funding to replace or compliment existing arrangements for primary care services.

• National workforce planning and analysis for primary health and community support services.

• A national evaluation, research and development program in primary health and community support services.

• National planning and priority setting processes for primary health and community care to ensure greater alignment of Commonwealth and State priorities.
